# Cloacal bacterial communities of tree swallows (*Tachycineta bicolor*): Similarity within a population, but not between pair-bonded social partners

**DOI:** 10.1371/journal.pone.0228982

**Published:** 2020-02-11

**Authors:** Jessica Hernandez, Camilo Escallón, Daniel Medina, Ben J. Vernasco, Jenifer B. Walke, Lisa K. Belden, Ignacio T. Moore

**Affiliations:** Department of Biological Sciences, Virginia Tech, Blacksburg, Virginia, United States of America; Universita degli Studi di Milano-Bicocca, ITALY

## Abstract

Host-associated microbial communities can influence the overall health of their animal hosts, and many factors, including behavior and physiology, can impact the formation of these complex communities. Bacteria within these communities can be transmitted socially between individuals via indirect (e.g., shared environments) or direct (e.g., physical contact) pathways. Limited research has been done to investigate how social interactions that occur in the context of mating shape host-associated microbial communities. To gain a better understanding of these interactions and, more specifically, to assess how mating behavior shapes an animal’s microbiome, we studied the cloacal bacterial communities of a socially monogamous yet genetically polygynous songbird, the North American tree swallow (*Tachycineta bicolor*). We address two questions: (1) do the cloacal bacterial communities differ between female and male tree swallows within a population? and (2) do pair-bonded social partners exhibit more similar cloacal bacterial communities than expected by chance? To answer these questions, we sampled the cloacal microbiome of adults during the breeding season and then used culture-independent, 16S rRNA gene amplicon sequencing to assess bacterial communities. Overall, we found that the cloacal bacterial communities of females and males were similar, and that the communities of pair-bonded social partners were not more similar than expected by chance. Our results suggest that social monogamy does not correlate with an increased similarity in cloacal bacterial community diversity or structure. As social partners were not assessed at the same time, it is possible that breeding stage differences masked social effects on bacterial community diversity and structure. Further, given that tree swallows exhibit high variation in rates of extra-pair activity, considering extra-pair activity when assessing cloacal microbial communities may be important for understanding how these bacterial communities are shaped. Further insight into how bacterial communities are shaped will ultimately shed light on potential tradeoffs associated with alternative behavioral strategies and socially-transmitted microbes.

## Introduction

Host-associated microbial communities can contribute to the overall health of their animal hosts [1–4], and many factors, including behavior and physiology, can impact the composition of these complex communities [1, 5–7]. Across animal taxa, for example, social behaviors, such as allopreening or allogrooming, cohabitation, group membership, and mating, play a role in shaping the diversity and structure of an animal’s microbial communities [8–13]. While microbes line most body surfaces, and distinct eco-evolutionary pressures may be imposed on each microbial community (e.g., those associated with the skin, feathers, oral, gut, cloaca) [14], the vast majority of studies relating social behavior and host-associated microbial communities have focused on the gut and skin [1].

In reptiles, amphibians, birds, and a few mammals the cloaca is the terminus for the digestive and reproductive tracts, and thus, cloacal microbial communities are impacted by both diet and reproductive behavior [10, 15]. Given that cloacal contact during copulation can result in the transmission of microbes between individuals [10] and that pair-bonded social partners maintain a relatively close relationship throughout the breeding season, it has been hypothesized that pair-bonded social partners share more similarity in their cloacal microbial communities compared to others in the same population [13, 16–19]. This hypothesis has been tested in several systems, and has generally been supported. For example, using culture-dependent bacterial assessment methods, Lombardo et al. [18] and Stewart and Rambo [19] showed similarity in a limited amount of pre-selected cloacal bacteria between social partners in tree swallows (*Tachycineta bicolor*) and house sparrows (*Passer domesticus*). More recent work using culture-independent methods has revealed that pair-bonded social partners experimentally blocked from making cloacal contact have significantly less similar cloacal bacterial communities compared to control pairs in black-legged kittiwakes (*Rissa tridactyla*) [13]. In addition, two observational studies on barn swallows (*Hirundo rustica*) in Europe found that cloacal bacterial communities of pair-bonded social partners were more similar to each other than to other individuals in the population or than expected by chance [16, 17]; however, the effect size was small in one of the studies [16].

To gain a more complete picture of the effects of social partnerships during the breeding season on cloacal bacterial community diversity and structure, and to follow up on prior studies in tree swallows [18] and barn swallows [16, 17], we used culture-independent, 16S rRNA gene amplicon sequencing to study the cloacal bacterial communities of breeding North American tree swallows. To our knowledge, this is the first study to apply culture-independent methods to study the cloacal microbiome of tree swallows. We asked two main questions: (1) do the cloacal bacterial communities differ between female and male tree swallows within a population? and (2) do pair-bonded social partners share more similar cloacal bacterial communities than expected by chance? For the first question, we hypothesized that the cloacal bacterial communities of females and males would differ due to physiological differences that exist between the sexes during the breeding season, such as differences in hormonal profiles [20] and immune activity [21]. For the second question, based on previous research examining the similarity of cloacal bacterial communities between social partners [13, 16–19], we hypothesized that cloacal bacterial communities between pair-bonded social partners would be more similar than expected by chance, because pair-bonded partners would interact more frequently with each other and with the same environment (i.e., the nest) than with other individuals in the population. Overall, this research used a sequencing-based approach to provide a more comprehensive assessment of the bacteria present within the cloacae of birds, as well as deepened our understanding of how social interactions, specifically mating partnerships, shape cloacal bacterial communities.

## Methods

### Study site and species

We studied a breeding population of free-living tree swallows (*Tachycineta bicolor*) at Kentland Farm, a 350-acre mosaic of fields owned by Virginia Tech, in Montgomery County, Virginia, U.S.A. (37°11’53.6 N, 80°34’58.0 W; 520 m.a.s.l.). Tree swallows are small, migratory, aerial insectivorous passerines [22]. They are socially monogamous and thus form pair-bonds during the breeding season that can extend across multiple years. Nevertheless, tree swallows exhibit high rates of extra-pair sexual activity (~50% of nestlings are extra-pair offspring), with both females and males engaging in extra-pair solicitations, copulations, and fertilizations [23, 24]. In our study population, tree swallows breed from late March to early August, and use man-made nest boxes set up along fence and tree lines that border open agricultural fields. The average distance between boxes occupied by tree swallows is approximately 25 meters. Within our study population, ~ 54% of the tree swallow offspring are from extra-pair fertilizations, with 84% of broods containing one or more extra-pair offspring (J. Hernandez unpublished data, based on 256 young and 55 broods).

### Sample collection

We captured adult tree swallows at nest boxes during the 2016 and 2017 breeding seasons from May to June. We caught the birds by hand when possible, otherwise we used a trap door to capture the birds when they entered the nest box. Upon capture, we banded each bird with a uniquely numbered aluminum U.S. Fish and Wildlife bird band. We also banded each female with a lime green color band and each male with a pink color band for visual identification from a distance. We identified females by the presence of a brood patch or, in the case of females in their first breeding season, by brown plumage. We identified males by the presence of an enlarged cloacal protuberance and the absence of a brood patch. By denoting the sex of each bird with a color band, we were able to target our capturing of each sex more effectively. Upon capture, we also measured the right wing and mass of each bird for subsequent host body condition analyses. To determine the pair-bonded social pair of a nest, we conducted observational surveys whereby we noted the female found to be incubating the eggs and the male seen to be feeding the subsequently hatched nestlings. We then sampled females during incubation and most males two to three weeks later during nestling provisioning. There was only one pair of the 13 total pairs we sampled in which the female and the male were sampled at the same time, since they were in the box copulating at the time of capture (J. Hernandez, personal observation). To collect cloacal bacteria, we gently inserted a sterile swab (PurFlock®, Puritan, USA) ~4 mm into the cloaca and revolved it once [25]. Swab samples were stored in sterile 1.5 mL tubes on ice in the field and later frozen in a -80°C freezer upon return to the lab (< 5 hours post-collection). We used gloves when handling and swabbing the birds, and changed gloves between individuals. The Virginia Tech Institutional Animal Care and Use Committee (IACUC) approved of this research.

### DNA extraction, amplification, and sequencing

To extract DNA from the cloacal swabs, we used the DNeasy Blood and Tissue Kit (Qiagen Inc., Valencia, CA, USA). We followed the manufacturer’s protocol for the purification of total DNA from animal tissues, including the pretreatment for gram-positive bacteria, for all swab samples. The pretreatment incorporates an additional incubation with lysozyme to most effectively lyse the cell walls of gram-positive bacteria prior to total DNA purification. We then targeted the hypervariable region 4 (V4) of the 16S rRNA gene for amplification using the primers 515F and 806R [26]. The reverse primer contained a GOLAY error-correcting 12bp barcode to tag individual samples. Similar to Costello et al. [27], we performed PCR in triplicate using 12 μL DNA-free PCR water (MO BIO, Carlsbad, CA, USA), 10 μL Hot MasterMix (5 PRIME, Gaithersburg, MD, USA), 0.5 μL of each forward and reverse primer, and 2 μL template DNA. We ran negative controls without template DNA for each sample. The thermal cycling conditions were as follows: 3 min at 94°C, 35 cycles with 45 s at 94°C, 1 min at 50°C, 1.5 min at 72°C, and 10 min at 72°C. Triplicate reactions were then pooled for each sample and visualized on a 1% agarose gel. We quantified the amplified bacterial DNA in each sample using a Qubit^TM^ 2.0 fluorometer and Qubit^TM^ dsDNA HS assay kit (Invitrogen, Carlsbad, CA, USA). Lastly, we pooled 200 ng DNA of each sample and purified the final pool (QIAquick PCR Purification kit, Qiagen, Valencia, CA, USA). This final pool was sent to the Dana Farber Cancer Institute of Harvard University to be sequenced on an Illumina MiSeq instrument, as described by Caporaso et al. [28], using a 250bp single-end strategy that captured most of the V4 region, which is 291bp. Samples collected from the 2016 and 2017 breeding seasons were extracted, amplified, and sequenced using identical protocols in August 2016 and 2017, respectively.

### Sequence data processing

Amplicon sequences for the 2016 and 2017 sequencing runs were combined for downstream data processing. Forward reads of raw Illumina 16S rRNA amplicon sequences were de-multiplexed and quality filtered using the Quantitative Insights Into Microbial Ecology (QIIME) 1.9 bioinformatics pipeline [29]. In QIIME, we removed sequences that contained errors in the barcode, and set the minimum number of consecutive high-quality base calls to include a read to 0.50 fraction of the input read length (default is 0.75). We also specified the minimum acceptable Phred quality score as 20. In other words, we kept the reads whose Phred score was equal to or above 20 to be of high quality and removed any reads below that threshold. Using the UCLUST method [30] and a 97% sequence similarity threshold, we then clustered sequences into operational taxonomic units (OTUs). We selected the most abundant sequence from each cluster to represent each OTU. We used PyNAST to search representative sequences against the Greengenes 13.8 database [31, 32]. The RDP classifier [33] was then used to assign the Greengenes taxonomy of each OTU using the default minimum confidence value of 0.5. OTUs with fewer than 0.01% of the total number of reads (i.e., fewer than 224 reads in our dataset) were removed [34]. Also, given that the sequencing depth per sample ranged from 9,360 to 119,903 reads per sample, we rarefied samples to 9,300 reads prior to analyses. Normalizing a dataset using rarefaction is appropriate, particularly when the sequencing depth across samples is highly variable and uneven, as is the case with our data set [35]. There was a 13-fold difference (i.e., highly uneven distribution) between the samples with the highest and lowest sequencing depths in our study.

### Statistical analyses

All analyses were performed in R (v. 3.4.1) [36]. To determine whether cloacal bacterial communities differed between adult female and male tree swallows, we assessed shared and unique OTUs between the sexes. In addition, we determined the most abundant OTUs in the cloacae of sampled birds using a relative abundance cut off of 5%. Abundant OTUs were identified to the minimum possible taxonomic level and the distribution of their relative abundance across samples was visualized using a heatmap (‘gplots’ package: function *heatmap*.*2*). To assess the bacterial diversity for each bird sampled (i.e., alpha diversity), we calculated OTU richness, the Shannon Index, and Faith’s phylogenetic distance in QIIME. Alpha diversity metrics of cloacal bacterial communities for both sexes were visualized using boxplots and generalized linear models were fitted to the diversity metrics to test for statistical differences between sexes. We used a negative binomial distribution with the log link function (‘MASS’ package: function *glm*.*nb*) for OTU richness to account for overdispersion (‘AER’: *dispersiontest*). We used a gamma distribution (‘MASS’: *glm*, family = Gamma) for the Shannon Index and Faith’s phylogenetic distance. In the generalized linear models, we included “Sex”, “Julian Date”, “Year”, and “Host Body Condition” as explanatory variables. To assess host body condition, we calculated a scaled-mass index based on a bird’s mass and wing length [37].

To assess whether variation in the structure of cloacal bacterial communities differed between females and males (i.e., beta diversity) in our population, we calculated non-parametric, permutational multivariate analyses of variance (PERMANOVA; ‘vegan’: *adonis*) based on 999 permutations. The results presented are based on Bray-Curtis and Jaccard metrics, because there were no apparent differences between results using Bray-Curtis or weighted UniFrac, or between Jaccard and unweighted UniFrac. Bray-Curtis and weighted UniFrac consider relative abundances, while Jaccard and unweighted UniFrac consider presence-absence. Bray-Curtis considers count-based data, while UniFrac metrics consider phylogenetic-based distances. Dissimilarity distances between females and males were then visualized using a non-metric multidimensional scaling (NMDS) plot. To compare the multivariate spread of the data between the sexes, we tested the multivariate homogeneity of group dispersions (‘vegan’: *betadisper*) using a permutation test (‘vegan’: *permutest*) for females and males.

To determine if the cloacal bacterial communities of pair-bonded social partners were more similar to each other compared to other randomly selected pairs, we compared the frequency distributions of sampled and randomized pairs within our study population. We manually calculated the difference between the OTU relative abundances of all possible combinations of hypothetical, randomly assigned female-male pairs. We took the absolute value of the OTU relative abundance differences and then generated a density plot to visualize the distribution of these differences for randomized female-male pairs. We repeated these steps to generate a density plot of the distribution of differences for sampled, pair-bonded female-male pairs. In a density plot, distributions that overlay each other generally indicate that the two distributions are not significantly different. To statistically compare the distributions of random and sampled pairs, and thus determine if sampled pairs were more similar to each other than expected by random chance, we performed a permutation test (‘coin’: *independence_test*). Further, we performed a hierarchical, polythetic, agglomerative cluster analysis based on Bray-Curtis and Jaccard distance metrics. This statistical method clusters together individuals with more similar cloacal bacterial communities; therefore, if pair-bonded social partners are clustered closer together, we can deduce that they have more similar cloacal bacterial communities compared to other birds in the study population.

## Results

We sampled a total of 13 social pairs (n = 4 pairs in 2016, 9 pairs in 2017), as well as an additional three females and four males for which we did not collect cloacal swab samples from their social partners (total n = 7 in 2016). Each bird was sampled only once. The average (± standard deviation) time between the sampling of female and male social partners was 14.23 (± 5.92) days, with the lowest and highest sampling time differences being 0 and 21 days, respectively. Only one social pair was sampled on the same day.

**Fig 1:**
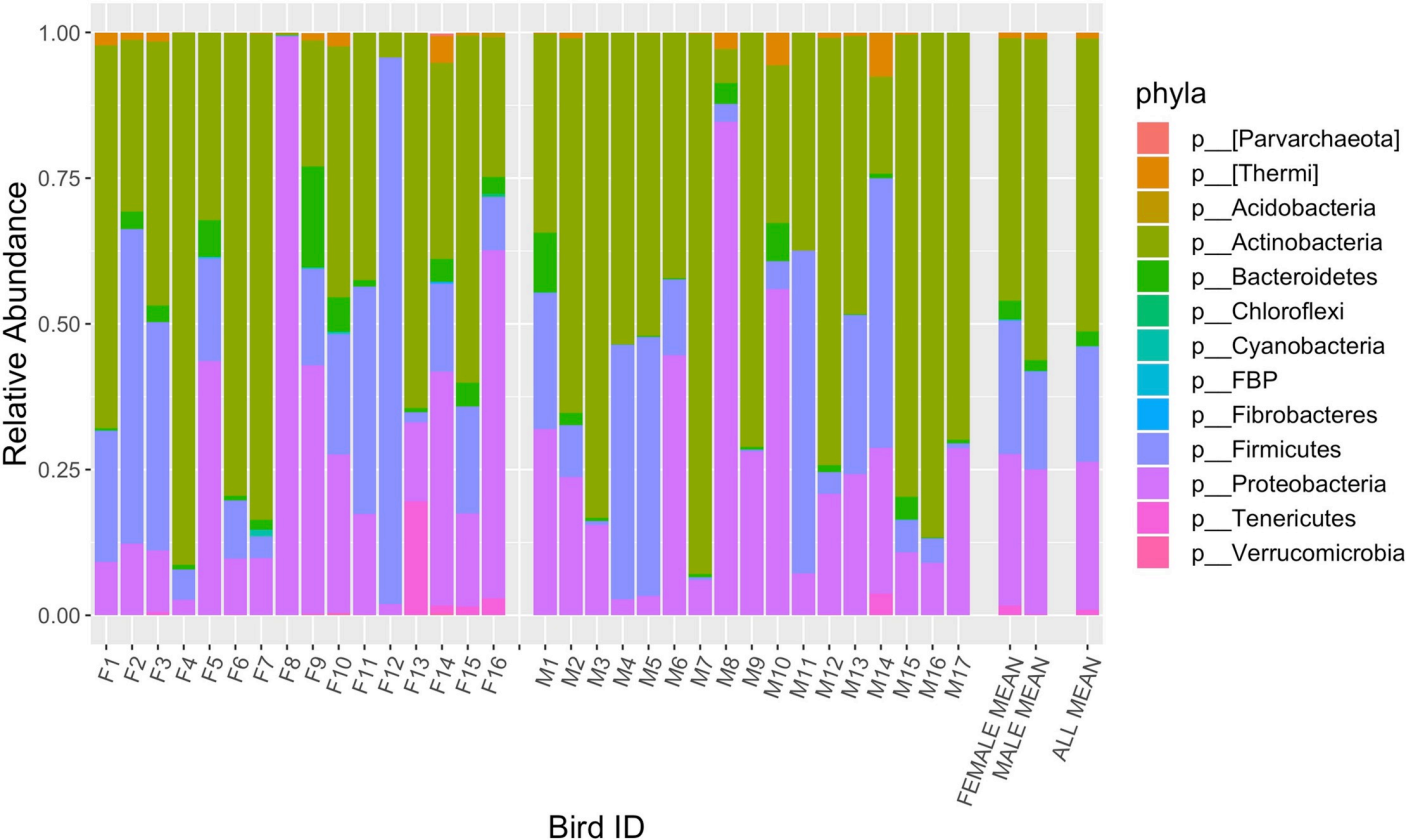
Mean relative abundance of bacterial phyla present in the cloacae of sampled adult tree swallows. Each column depicts the phyla represented within the cloaca of an individual bird. ‘F’ and ‘M’ refer to individually sampled females and males, respectively. ‘Female Mean’ and ‘Male Mean’ depict the average phyla represented per sex. ‘All Mean’ depicts the average phyla represented across sexes.

**Fig 2:**
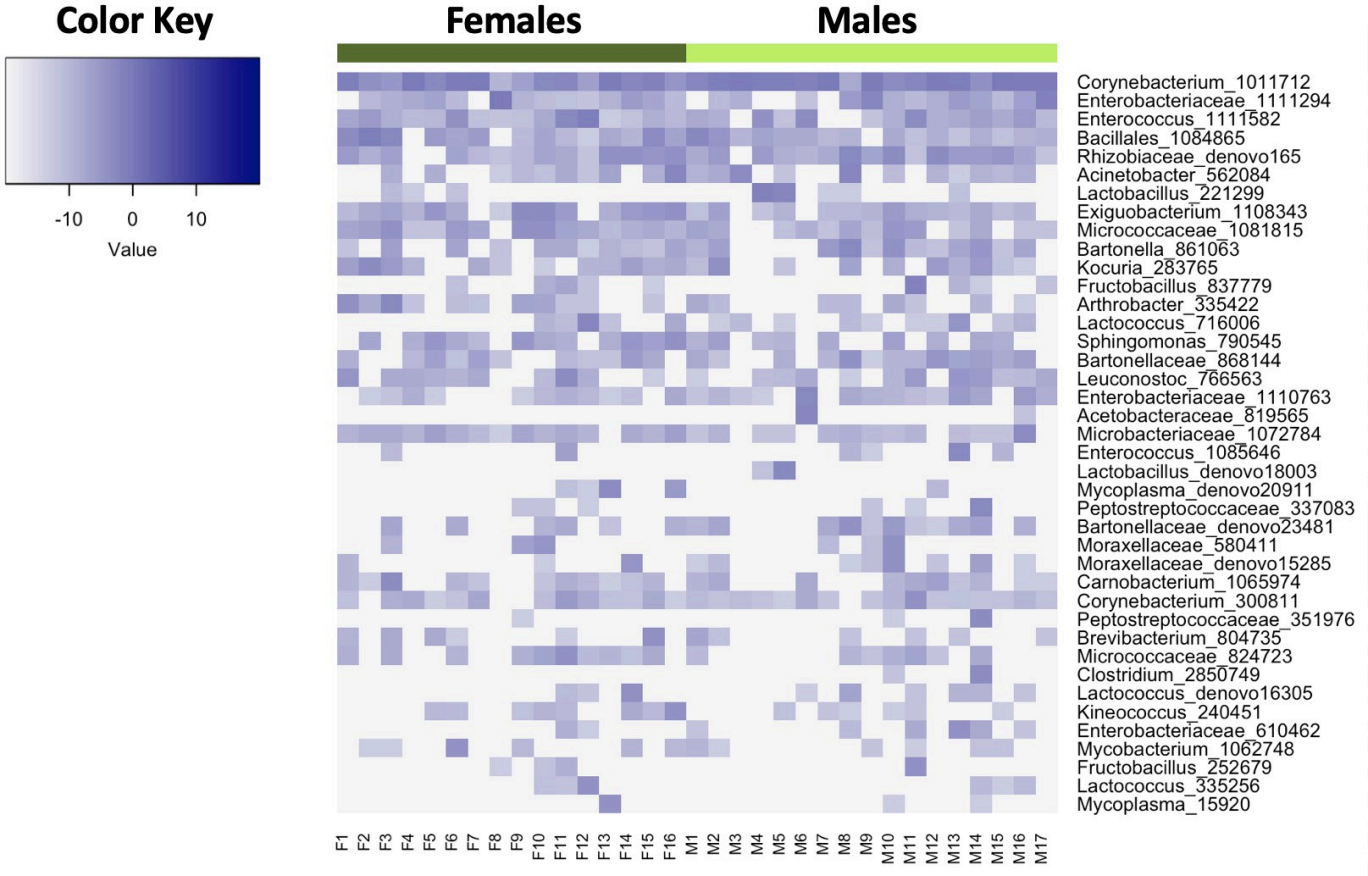
Heatmap of relative abundances (log-transformed) of the most abundant OTUs present in the cloacae of adult tree swallows. Each column depicts individual birds sampled. ‘F’ and ‘M’ refer to individually sampled females and males, respectively. The dark green bar at the top highlights sampled females, while the light green bar highlights sampled males. Each row depicts a bacterial taxon present at a relative abundance greater than 5% in the sampled population. Bacterial taxa are labeled to the lowest possible level of taxonomic classification.

We identified 594 OTUs across the 33 individual bird cloacal samples. The bacterial phyla with the highest relative abundances were Actinobacteria, Firmicutes, and Proteobacteria (Fig 1). The most prevalent family, Corynebacteriaceae (phylum Actinobacteria), was present in 100% of individuals, followed by the families Microbacteriaceae (phylum Actinobacteria), Rhizobiaceae (phylum Proteobacteria), and Enterococcaceae (phylum Firmicutes), which were present across at least 90% of individuals. Only OTUs from the genus Corynebacterium (phylum Actinobacteria, class Actinobacteria, order Actinomycetales, family Corynebacteriaceae) comprised the core microbiome, which were present in the cloacae of 100% of birds sampled, and occurred at a relative abundance of ~40% across individuals (Fig 2). OTUs from the families Enterobacteriaceae (phylum Proteobacteria, class Gammaproteobacteria, order Enterobacteriales) and Micrococcaceae (phylum Actinobacteria, class Actinobacteria, order Actinomycetales) were the second and third most abundant and were each present across individuals at mean relative abundances of ~7.5 and 4.7%, respectively (Fig 2).

**Fig 3:**
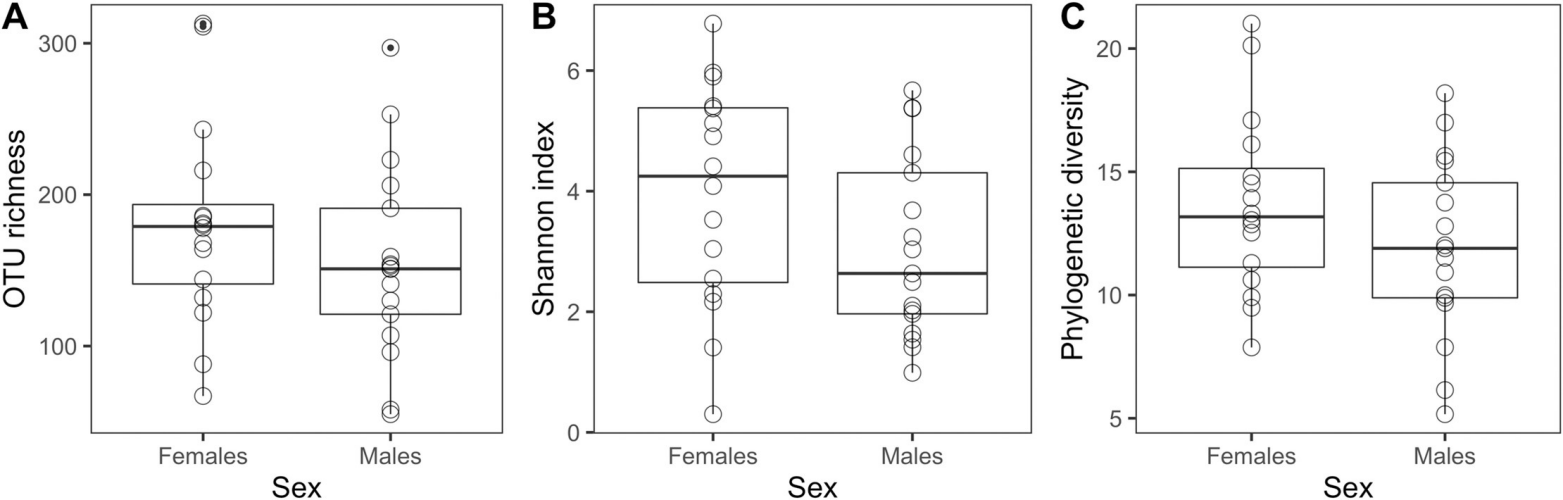
Alpha diversity metrics for bacterial OTUs sampled from the cloacae of adult tree swallows. (A) OTU richness (i.e., observed OTUs), (B), Shannon Index, and (C) Faith’s phylogenetic diversity indices were calculated. The circles represent individuals sampled of each sex.

**Fig 4:**
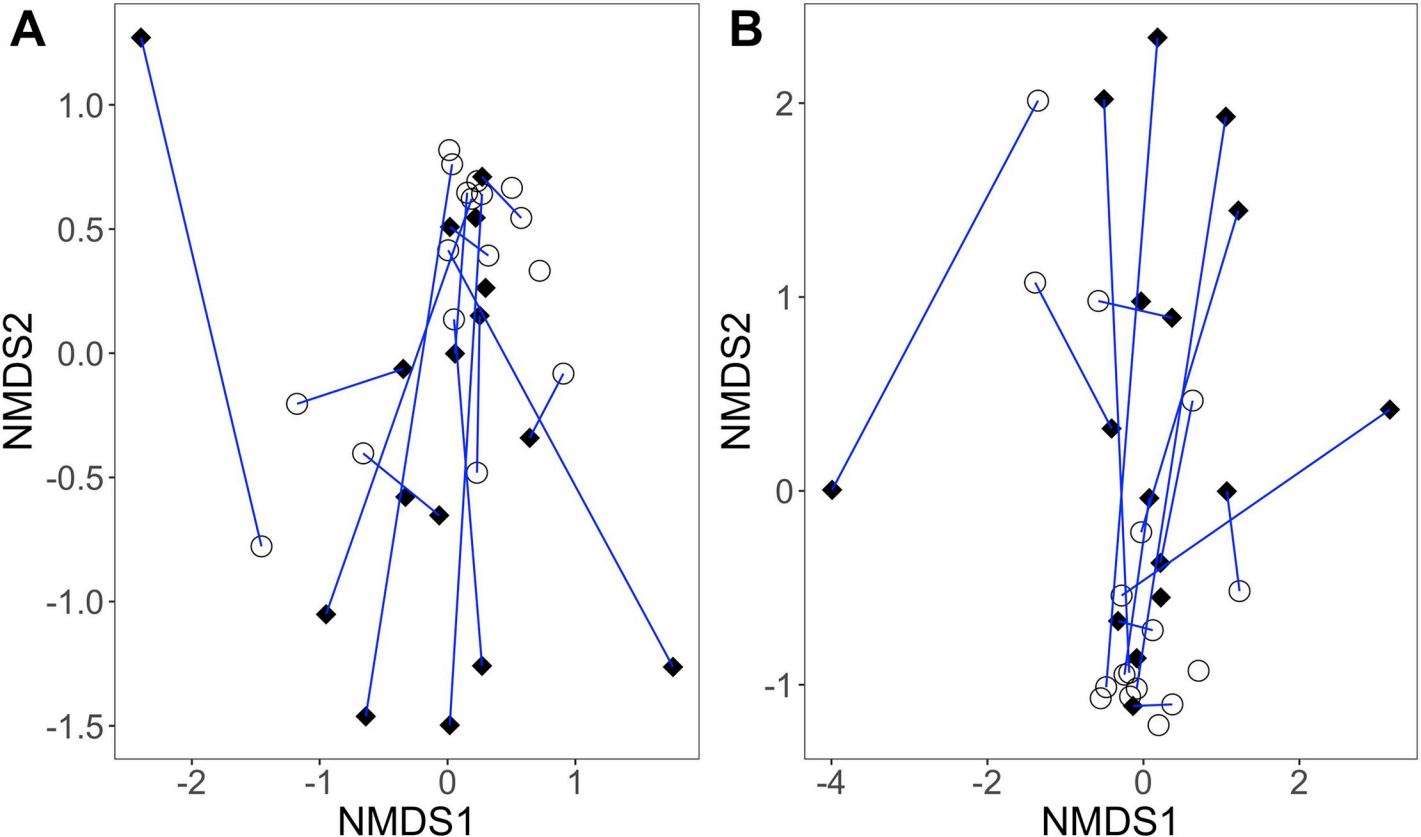
Cloacal bacterial community structure of adult tree swallows. Non-metric multidimensional scaling (NMDS) ordinations are based on (A) Bray-Curtis and (B) Jaccard distance metrics. Each symbol represents one individual (black diamonds = females, open circles = males). Pair-bonded social partners are connected by a blue line. Points closer together exhibit individuals with more similar cloacal bacterial community structure.

Females and males had similar OTU richness (*glm*.*nb*, b = 0.041, SE = 0.19, z_(29)_ = 0.21, p = 0.83), with an average (± standard deviation) of 179 ± 67 OTUs in females and 155 ± 63 OTUs in males. There was also not a significant difference between the bacterial communities of females and males in terms of the Shannon Index, which considers both richness and evenness of the community (*glm*, b = 0.031, SE = 0.072, t_(29)_ = 0.42, p = 0.68) or Faith’s phylogenetic diversity (*glm*, b = 0.0065, SE = 0.012, t_(29)_ = 0.55, p = 0.59) (Fig 3). Additionally, there were no differences in cloacal bacterial community diversity within seasons (*glm*.*nb*, OTU richness: b = -0.012, SE = 0.0077, z_(30)_ = -1.5, p = 0.13, *glm*, Shannon: b = -0.055, SE = 0.034, t_(30)_ = -1.6, p = 0.12, *glm*, Faith’s: b = -0.084, SE = 0.070, t_(30)_ = -1.2, p = 0.24) or between seasons (*glm*.*nb*, OTU richness: b = 0.19, SE = 0.13, z_(30)_ = 1.4, p = 0.15, *glm*, Shannon: b = 0.55, SE = 0.59, t_(30)_ = 0.94, p = 0.36), with one exception (i.e., *glm*, Faith’s: b = 2.6, SE = 1.2, t_(30)_ = 2.1, p = 0.040). There was not a significant relationship between cloacal bacterial community diversity and host body condition (*glm*.*nb*, OTU richness: b = 0.024, SE = 0.049, z_(30)_ = 0.50, p = 0.62, *glm*, Shannon: b = -0.025, SE = 0.017, t_(30)_ = -1.5, p = 0.15, *glm*, Faith’s: b = -0.0017, SE = 0.0028, t_(30)_ = -0.61, p = 0.55). Further, females and males did not significantly differ in cloacal bacterial community structure (*adonis*, Bray-Curtis: pseudo-F_(1,31)_ = 0.77, R^2^ = 0.024, p = 0.68; Jaccard: pseudo-F_(1,31)_ = 0.84, R^2^ = 0.026, p = 0.66) (Fig 4). The multidimensional spread of female and male cloacal bacterial communities was not significantly different (*permutest*, Bray-Curtis: F_(1,31)_ = 1.9, p = 0.18; Jaccard: F_(1,31)_ = 1.8, p = 0.18); thus, there was no statistical evidence for a difference in dispersion between the cloacal bacterial communities of each sex.

**Fig 5:**
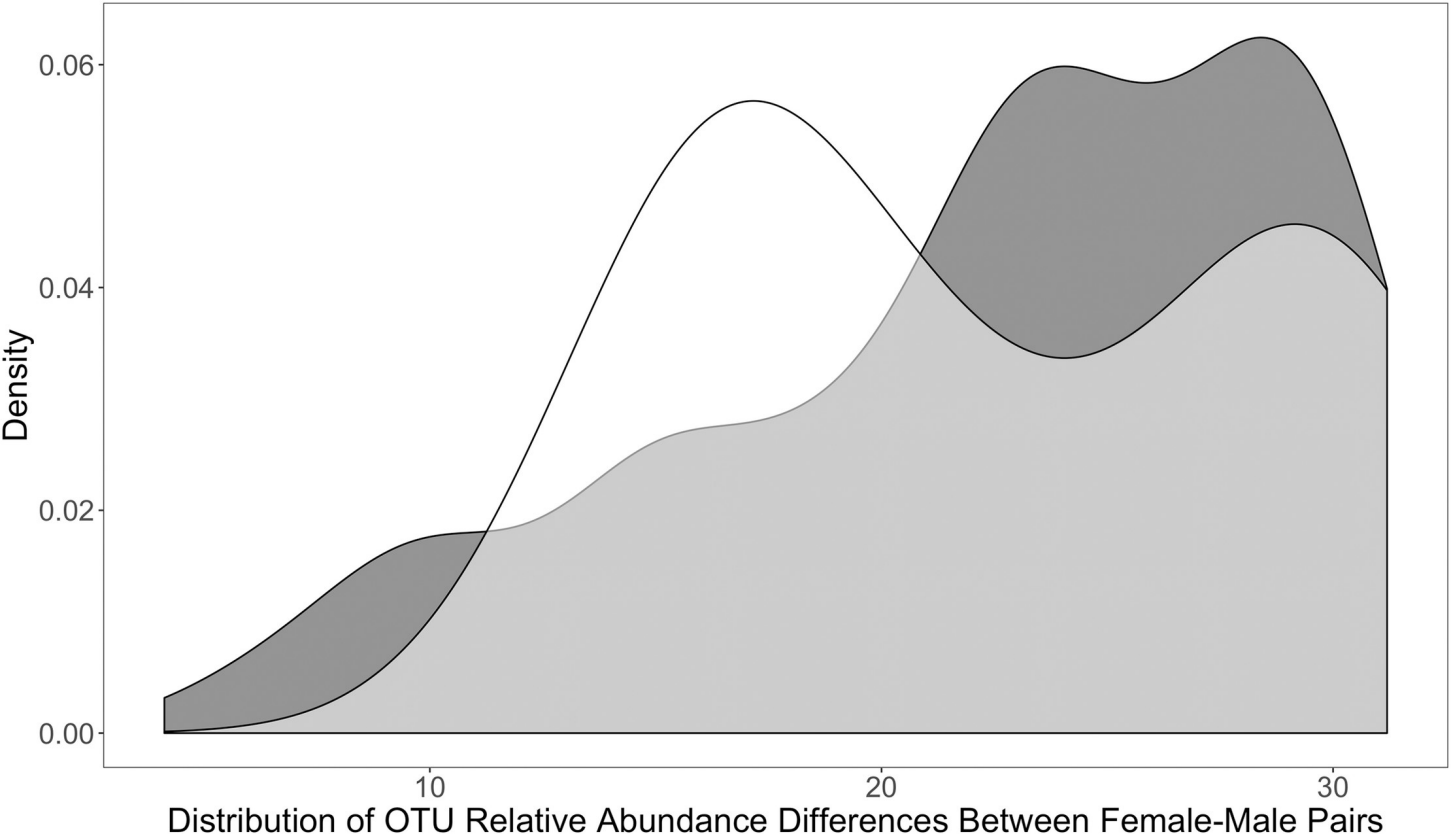
**OTU relative abundance differences for randomized (dark gray) and sampled (white) female-male pairs.** Samples on the left side of the distribution are more similar, while samples on the right side are less similar and thus exhibit greater differences.

**Fig 6:**
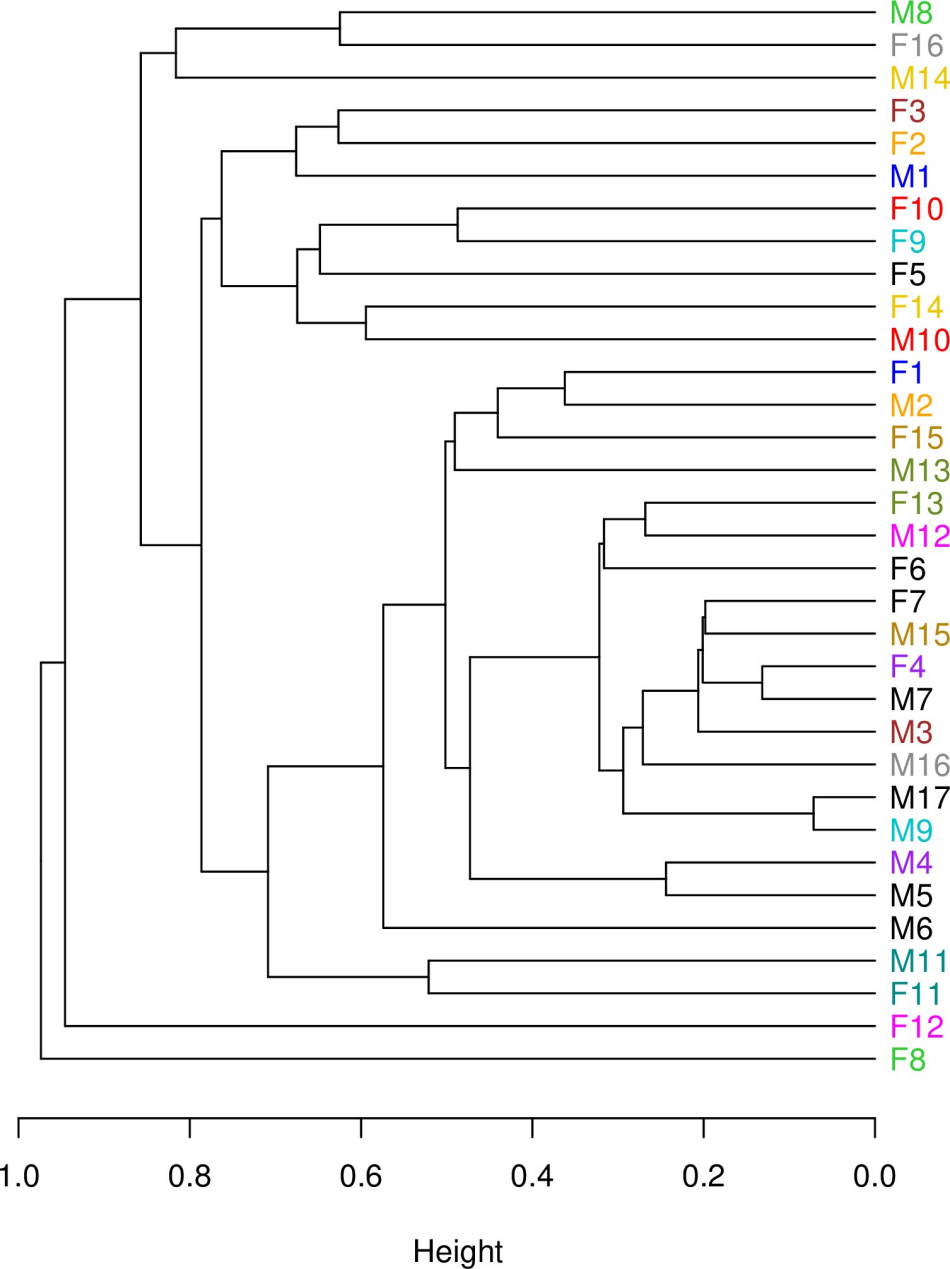
Hierarchical cluster analysis (based on Bray-Curtis dissimilarity) showing the overall level of similarity of cloacal bacterial communities for sampled females and males. The cluster analysis using the Jaccard distance metric was visually indistinguishable from the Bray-Curtis based analysis, so we only show the latter here. ‘F’ = female, ‘M’ = male, similar color = social pair, black = individual was sampled but not its social partner.

The difference in cloacal bacterial composition between sampled pair-bonded female-male social pairs was not statistically different from the difference in cloacal bacterial composition between randomly paired female-male pairs within our study population (*independence_test*, Z = 0.039, p = 0.97) (Fig 5). Additionally, based on the composition of their cloacal bacterial communities, pair-bonded social partners did not cluster more closely than other sampled individuals (Fig 4, partners are connected by a blue line; Fig 6). In other words, social partners did not have more similar cloacal bacterial communities than other sampled individuals. Only the one social pair that we sampled at the same time exhibited cloacal bacterial communities more similar to each other than to other individuals in the population (Fig 6, see ‘F11’ and ‘M11’).

## Discussion

In this study, we assessed (1) whether the cloacal bacterial communities differed between female and male tree swallows within a population, and (2) whether pair-bonded social partners shared more similar cloacal bacterial communities than expected by chance. Using 16S rRNA gene amplicon sequencing, we found that cloacal bacterial communities were generally similar between female and male tree swallows. We also found that the cloacal bacterial communities of pair-bonded social partners were not more similar than expected by chance. That is, cloacal bacterial community diversity and structure did not vary based on sex or social pair bond.

Neither of our hypotheses were supported and this could be due to rates of extra-pair copulations and/or diet. First, birds transmit bacteria via copulations through both cloacal contact and ejaculate transfer [10, 38] and high rates of extra-pair copulations could serve to homogenize the cloacal bacterial communities across the population [16]. Second, it is important to bear in mind that the cloaca functions as the terminus for both the reproductive and the digestive tract in birds. The role that diet plays in shaping the cloacal bacterial communities of birds is currently unclear, however, previous research has found that diet shapes the bacterial communities anterior to the cloaca (e.g., the crop, small and large intestines, and cecum) across bird species [39]. Previous work on breeding tree swallows has found that adults forage near their breeding site and tend to consistently select similar prey items [40]. Given that the tree swallows sampled in this study likely forage in the same general area on the same available prey items, we would expect diet composition to be similar across birds in our study population. Thus, future studies trying to understand the factors that shape cloacal bacterial communities should primarily consider rates of extra-pair copulations.

Among birds, the behavior and physiology of both sexes change as the breeding season progresses (e.g. [41–43]), and these behavioral and physiological changes may also influence the diversity and structure of cloacal bacterial communities. In this study, we sampled pair-bonded female and male social partners during different stages of the breeding season (i.e., females during incubation and most males several weeks later during nestling provisioning). There was only one pair in which the female and the male were sampled at the same time, since they were in the box copulating at the time of capture. Interestingly, only this pair-bonded pair exhibited cloacal bacterial communities that were more similar to each other than to other individuals in the population or than expected by chance. Therefore, it is possible that partners exhibit similarity in their cloacal bacterial communities, but detecting this similarity requires sampling of social partners at the same time or, at the very least, during the same breeding stage. For example, in previous studies focused on socially monogamous yet genetically polygynous birds, both females and males were sampled during nestling provisioning and found to have similar cloacal bacterial communities [16; 18–19]. However, of these previous studies two [18,19] were culture-dependent studies that focused on a limited amount of pre-selected bacteria, and the other [16] found a small effect with regard to females and males having similar cloacal bacterial communities. In a study focused on a truly monogamous bird, the black-legged kittiwake (*Rissa tridactyla*), females and males of a social pair were sampled during incubation and nestling provisioning, respectively, and were nevertheless found to have similar cloacal bacterial communities [13]. Given that black-legged kittiwakes exhibit true monogamy, such high fidelity between social partners may have maintained high similarity between the cloacal bacterial communities of pairs, regardless of females and males being sampled during different stages of the breeding season. Overall, to account for any changes in behavior and physiology across the breeding season and to most effectively assess similarity in cloacal bacterial communities between social partners, the sampling of both partners should be coordinated to occur at the same time.

Previous work examining the similarity of the cloacal bacteria of social partners in tree swallows used culture-dependent methods [18]. The isolates cultured and studied by Lombardo et al. (see Table 1 in [18]) included Lactobacilli, *Staphylococcus* spp., *Campylobacter* spp., *Salmonella* spp., and *Shigella* spp. In our study, we used culture-independent, next-generation sequencing. We did not find that any of the cloacal bacteria they cultured were members of the core bacterial communities of birds within our study population. While *Staphylococcus* spp. and Lactobacilli were detected, their relative abundances were <0.001%. This suggests that culturing of cloacal bacteria may not consistently result in isolation of the most dominant bacteria in the system. It is also possible that the bacteria cultured by Lombardo et al. [18] are rare in our study population and these bacteria were not retained during sample processing or sequence quality filtering.

In conclusion, our results suggest that the cloacal bacterial communities of female and male tree swallows are similar within our study population and that pair-bonded social partners do not share more similar cloacal bacterial communities than expected by chance. Given that tree swallows exhibit high variation in rates of extra-pair activity, we argue that considering rates of extra-pair activity or the number of sexual partners per bird when assessing cloacal bacterial communities may be important for understanding how cloacal bacterial communities are structured. Also, since cloacal bacterial communities comprise bacteria derived from both the reproductive and digestive tract, diet should also be considered when assessing cloacal bacterial communities. Lastly, we suggest that pair-bonded social partners should be sampled simultaneously to control for any temporal shifts in individual behavior and physiology that may influence shifts in cloacal bacterial community structure across breeding stages.
